# Food bank operational characteristics and rates of food bank use across Britain

**DOI:** 10.1186/s12889-019-6951-6

**Published:** 2019-05-14

**Authors:** Rachel Loopstra, Hannah Lambie-Mumford, Jasmine Fledderjohann

**Affiliations:** 1 Department of Nutritional Sciences, Faculty of Life Sciences and Medicine, King’s College London, 150 Stamford Street, Franklin-Wilkins Building, London, SE1 9NH UK; 2 Sheffield Political Economy Research Institute (SPERI), Faculty of Social Sciences, University of Sheffield, ICoSS Portobello, Sheffield, S1 4DP UK; 3 Department of Sociology, Lancaster University, Bowland North, Lancaster, LA1 4YN UK

**Keywords:** Food insecurity, Food banks, Food pantries, Disability, Food access

## Abstract

**Background:**

Food banks are a common community-based response to household food insecurity in high-income countries. While the profile of their users and nature of the quality of food they provide have been researched, few studies have examined their operational characteristics to explore the accessibility of their services for people at risk of food insecurity. This study describes the nature of operations in a food bank network operating in Britain and explores how operations are associated with volume of use.

**Methods:**

Data from The Trussell Trust Foodbank’s network of 1145 distribution centres in 2015/16 on hours of operation, locations, and usage were combined with national statistics on Working Tax Credit claimants, disability and unemployment. Descriptive statistics focused on how often and when food banks were open within local authorities. The relationships between operational characteristics and volume of use were examined using regression analyses. Interaction terms tested how relationships between indicators of need with food bank usage changed with operational characteristics.

**Results:**

Weekday operating hours were primarily between the hours of 10 a.m. and 2 p.m., but at any given hour no more than 20% of distribution centres were open, with fewer than 3% open after 4 pm. Where food banks had fewer distribution centres and operating hours, the volume of food bank usage was lower. In-work poverty, disability, and unemployment rates were all associated with higher volume of usage; however, the relationship between disability and food bank use was modified by the density of food banks and number of operating hours. Where food banks were less accessible, the relationship between disability and food bank use was diminished.

**Conclusions:**

These findings suggest operational characteristics are an important part of access to food banks and raise questions about the ability of food banks to meet the needs of people at risk of food insecurity in Britain.

**Electronic supplementary material:**

The online version of this article (10.1186/s12889-019-6951-6) contains supplementary material, which is available to authorized users.

## Background

Household food insecurity—that is, insecure or insufficient access to food arising from a lack of financial resources— affects up to 21% of adults or households in some high-income countries [1–4]. Recent data from England, Wales, and Northern Ireland suggest 8% of adults experience moderate or severe food insecurity, while an additional 13% report marginal experiences [5]. Food insecurity is associated with a number of negative social and health consequences [6], including depression and anxiety [7], elevated risk and poor management of chronic diseases [8–11], and poor child health [12].

In times of economic downturn and welfare retrenchment, charities often expand in an attempt to address food insecurity in local communities [13]. These responses frequently take the form of food banks (in the US, food pantries)-- places where parcels of groceries can be picked up for home consumption. In the US and Canada, food banks began to expand through the 1980s, with a further expansion in the mid-1990s during major cut-backs and restructuring to the welfare state [14, 15]. In the UK, food banks were rare until 2010, when The Trussell Trust Foodbank Network, then a social franchise of networked food banks, expanded rapidly. The expansion of Trussell Trust food banks and their use has been linked to local authority budget cuts, welfare reforms, and reduced welfare entitlements [16–19].

There is now a large body of literature exploring whether food banks can reduce food insecurity in high-income countries [20–22]. From a supply-side perspective, there are two crucial requirements for food banks to be able to reduce food insecurity: they must provide a quantity and quality of food to meet the nutritional and food security needs of their clients, and they must be accessible to people who experience food insecurity. On the former, previous studies have found high risk of dietary inadequacies and high levels of severe food insecurity among food bank users [23], and food banks have been found to provide an inadequate supply of dairy foods, and insufficient amounts of calcium and vitamins A and C, in the food parcels provided [24]. Many studies have also highlighted that food banks are unable to provide a healthy balance of foods, relying heavily on non-perishable goods [23]. Tarasuk et al. also found that many food banks operating in Canada regularly had to reduce the amounts of food they give out and some had to turn people away [25]. Together, these studies suggest food banks are limited in their ability to provide the quantity and quality of food needed to address the food insecurity and nutritional vulnerability of their clients.

As above, the second critical element of how effectively food banks can address food insecurity is their accessibility, but few studies have examined this. Based on data from one food bank provider in California (Second Harvest) and corresponding data for one county, Bacon and Baker [26] used GIS to map the relationships between food distribution locations and an approximation of area-level food insecurity (based on poverty rates, unemployment, and tenancy data). While a majority of areas estimated to have high food insecurity rates had good access to food distribution sites, 42% had low access (defined as 67% or more of the census tract being outside a 1-mile buffer to a distribution location). Research from the UK has shown food banks are more likely to be located in local authorities with higher rates of unemployment and child deprivation [27], but another study found that Trussell Trust ‘Foodbank’ locations (referring to the network membership entity that coordinates food parcel distribution across their various distribution sites) were not associated with other potential indicators of need, namely welfare caseloads [28].

However, geographical location is only one aspect of access. Based on qualitative interviews with food bank managers and employees from The Trussell Trust Foodbank Network, which is the largest network of food banks in Britain, Lambie-Mumford raised concerns that the operational characteristics of food banks may also inhibit access [13]. These include the use of referrals, whereby gatekeeper agencies determine eligibility for vouchers to the food bank; suggestions to restrict the number of times people can receive referrals (e.g. no more than three times in 6 months at the discretion of food bank managers); and limited operating hours. Similarly, in Canada, a survey of food banks in five cities showed that most only operated one or two days per week, with only 8.5% reporting being open on weekends [25].

To our knowledge, whether there is a quantitative link between operational characteristics and volume of usage at different food banks has not been explored. Here, using data on two metrics of access (hours of operation and number of food banks in local areas), we explore the relationship between volume of food bank use and food bank operations in the largest food bank network operating in Britain. We focus on two key questions: First, how does access to food banks vary in terms of geography and hours of operation? Here, we describe the variation in density of food banks across local areas and their hours of operation. Second, we ask, how do variation in food bank access and local authority characteristics jointly relate to the number of food parcels distributed? On this question, we explore two hypotheses. First, we hypothesise that better accessibility (in terms of operational characteristics, i.e. geographic distribution and operating hours) of food banks is positively associated with the number of food parcels distributed in local authorities. Second, drawing on previous evidence that unemployment, in-work poverty, and disability are associated with food bank use, we test if poor access to food banks moderates these associations. Specifically, we test if the relationships between the level of food parcel distribution and these indicators of need are modified by the density of food bank distribution centres in local areas or hours of food bank operation. We discuss these hypotheses in greater detail below.

## Methods

### The Trussell Trust Foodbank Network

We conduct our analysis using data from The Trussell Trust Foodbank Network. The Trussell Trust was established as a social franchise in 2004, enabling Christian churches and community groups to join their network and replicate their specific model (called a ‘Foodbank’) [13, 29]. Within The Trussell Trust, a Foodbank refers to the membership entity, which may involve only one church or a number of churches or Christian groups. The Foodbank coordinates food distribution and operations in their catchment area. It may have only one distribution site (a place where people redeem their referral voucher and pick up their food parcel) or multiple distribution sites run by different churches. Some also run a mobile delivery service or provide parcels for pick-up from referring agencies. It is food bank distribution centres, however, that are equivalent to what is commonly referred to as food banks or food pantries in the research literature [20]. Hereafter, we refer to outward-facing sites for food parcel distribution as food banks/distribution sites, while Foodbanks refer to the Trussell Trust coordinating entity. The Trussell Trust runs about 1235 distribution sites [30]--about 60% of all food banks in the UK [31]. It is the only national network of food banks where members operate their food banks according to common guidelines and collect harmonised data on usage.

Like those in other countries, Trussell Trust food banks provide a parcel of mostly non-perishable foods, free of cost, to people seeking their assistance. People may pick up one food parcel per household. The Trussell Trust provides guidance on what each parcel should contain and the amount of food provided is adjusted based on the number of people in the household. The Trussell Trust specifies the amount as three days’ worth of nutritionally-balanced food (~ 10 meals) [29]. Trussell Trust food banks require that individuals first obtain a referral voucher from a frontline care or health professional. Referral vouchers are held by local community organisations with whom food banks have established relationships, and thus can vary from area to area. They can include welfare services, local authorities, Citizens Advice Bureaux, GP practices, social workers or schools [30].

### Data

Trussell Trust food bank data for fiscal year 2015/16 were provided. The Trussell Trust collects data on volume of usage by tracking the number of times that people benefit from their food parcels. They do not count the unique number of people or households receiving food parcels. Instead, they count the number of people helped by each referral voucher redeemed in their food banks, i.e. the number of adults and children in the household issued the voucher.^1^ These data are referred to as “food bank usage” throughout the paper and when described empirically, as “instances of people receiving help from a food parcel”, since they do not describe unique beneficiaries.

These data were combined with a database maintained by The Trussell Trust on Foodbank locations and hours for distribution sites. We coded dichotomous indicators for whether distribution sites were operating on weekends (any hours on Saturday and/or Sunday) and, separately, whether they were operating in evenings (i.e. after 6 pm).

These data were then linked to area-level data for the local authority in which they were located. This involved summing across all food bank distribution centres in a given local authority to result in aggregate figures for food parcel distribution and operations at the local authority level. Local authorities refer to the 380 district councils, Unitary Authorities, Metropolitan Areas, and London Boroughs in England, Scotland, and Wales. The specific local authority data we linked food bank data to were 2015/16 working-age unemployment rates, Equality Act Core disability and work-limiting disability rates, and Working Tax Credit claimants (an indicator of in-work poverty). These data were available from nomisweb.co.uk and HM Revenue and Customs [32].

Compiling these data resulted in a dataset of food bank usage in 259 local authorities for 2015/2016 (Additional file 1). There were a total of 101 local authorities in which Trussell Trust Foodbanks did not operate by in 2015/16 (just over one quarter of the local authorities in England, Scotland and Wales). We also excluded 15 local authorities where food banks operated, but for which there were no data in 2015/16, and 5 local authorities with small populations because they do not have reliable area-level data.

### Analysis

In our statistical models, we tested three hypotheses:

1. Longer food bank opening hours and greater food bank density in local authorities are positively associated with the volume of food bank usage in local authorities.
2. Disability, in-work poverty, and unemployment will be more strongly related to food bank usage where food banks are open more hours and where there is a greater density of food banks.
3. In-work poverty will be more strongly related to food bank usage in areas where food banks are open on weekends and on evenings.

These hypotheses are rooted in previous research showing households using food banks have extremely low incomes [33, 34], and that low income drives food insecurity [35]. However, compared to the low-income population overall in the UK, some groups are over-represented in food banks and some are under-represented [33]. Specifically, unemployed adults and those with disabilities are over-represented in food banks; adults who are working are under-represented.

We first used descriptive statistics and graphics to visually examine the nature of food bank operations. To test our first hypothesis, linear regression models were used to examine how operational characteristics related to total food bank use over 2015/16. Operational characteristics included whether or not any food banks operated on the weekends or in the evenings in the local authority, the number of food bank distribution sites, and land area of the local authority. We excluded two local authorities that had extreme values for hours of operation (>99th percentile), as these were likely food parcel pick-up sites that are open all day rather than food bank distribution sites.

To test our remaining hypotheses, interaction terms between operational characteristics and predictor variables were included in regression models. Where significant interaction terms were observed, margins plots were used for visualisation. Corresponding regression coefficients for main effects and interactions underlying the figures are presented in Additional file 1. All analyses were carried out with Stata 15.

## Results

In 2015/16, among the active 392 Trussell Trust Foodbanks in England, Scotland, and Wales, there were 1145 food bank distribution sites operating. Foodbanks operated an average of 2.89 distribution sites, but this ranged from 1 to 23, with nine Foodbanks operating more than 10 sites. At the local authority level, about 60% had 6 or more distribution sites. After accounting for land size, the average number of distribution sites in local authorities was 3.42 per 100 km^2^ (SD = 4.95) (Table 1).

**Table 1:** Descriptive characteristics for local authorities with food banks (2015/16)

Variable	Local authorities	Mean or %	Std. Dev.	Min	Max
Food parcel distribution as percent of population	257	2.37	1.63	0.13	13.2
Number of day-time hours food banks open in local authority per week	257	16.4	17.9	0	158.5
Number of evening hours food banks open in local authority per week	257	0.28	0.93	0	10
Number of weekend hours food banks open in local authority per week	257	0.6	1.28	0	6
Distribution sites per 100 KM^2^	257	3.43	4.95	0.02	27.5
Equality Act core disabled and work-limited disabled rate (% working-age adults)	257	19.50%	4.4	8.5	33
Unemployment rate (% working-age adults)	233	5.20%	1.96	1.7	11.1
Working Tax Credits (% households)	257	10.90%	3.13	4.07	23.6
Any food banks open on weekends					
No	203	78.99%	0.41	0	1
Yes	54	21.01%	0.41	0	1
Any food banks open in evenings					
No	223	86.77%	0.34	0	1
Yes	34	13.23%	0.34	0	1
Daytime opening hours					
Less than 15 h per week	162	63.78%	0.48	0	1
15 h + per week	92	36.22%	0.48	0	1
Food bank density					
Less than 1 per 100 KM^2^	114	44.36%	0.50	0	1
1–2 per 100 KM^2^	63	24.51%	0.43	0	1
3+ per 100 KM^2^	80	31.13%	0.46	0	1

**Fig. 1:**
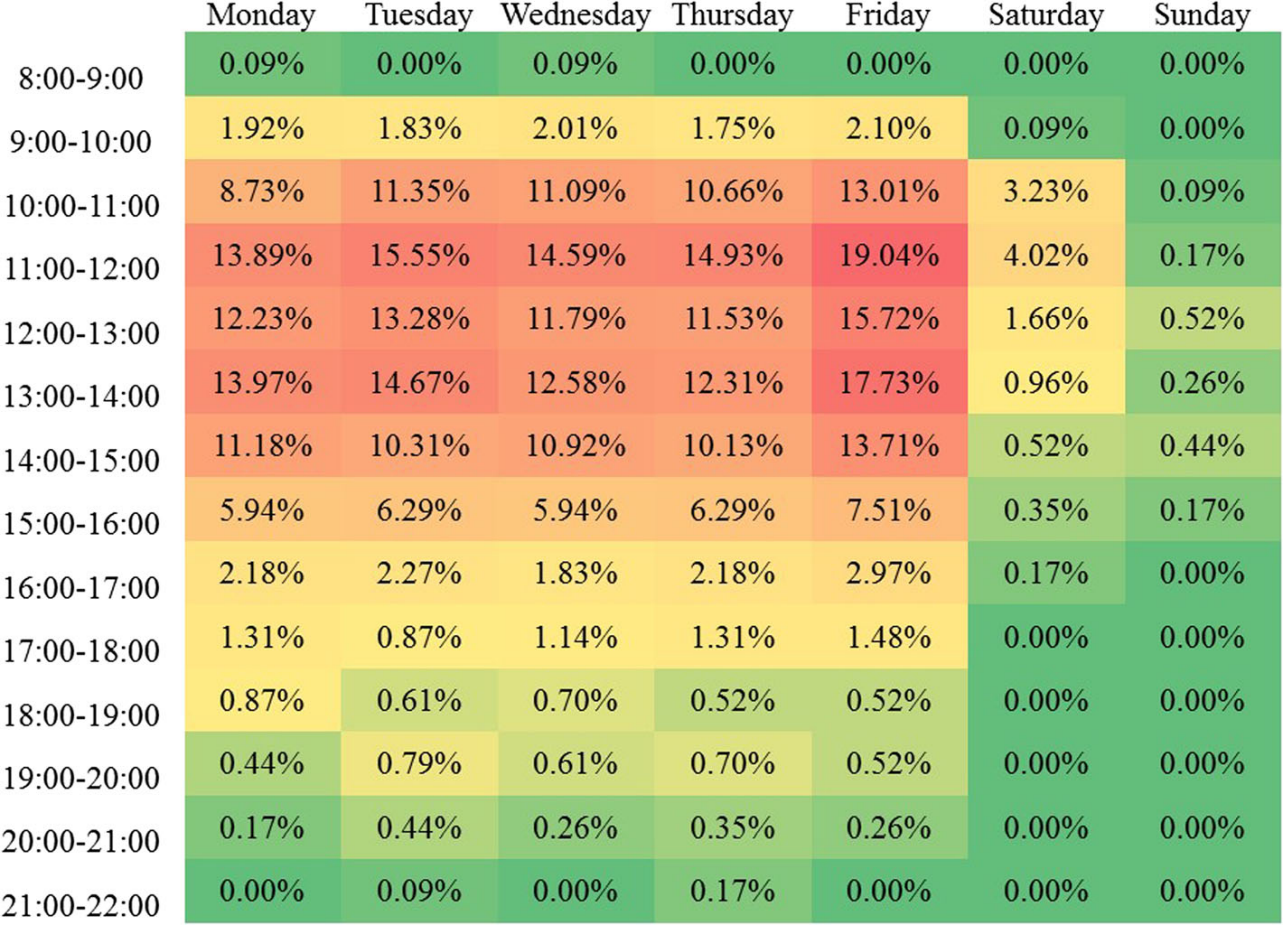
Frequency of food bank opening hours as a proportion of the total number of food banks that could operate

Figure 1 shows a heat map highlighting when food bank distribution centres were open during the week. Fewer than 20% were open in any given hour of the week. Hours were not evenly spread during the week, with operating hours concentrating between the hours of 10:00 a.m. and 2:00 p.m. Very few sites were open at any given time in hours after 4:00 p.m. Similarly, very few food banks were operating in any given hour on weekends.

**Fig. 2:**
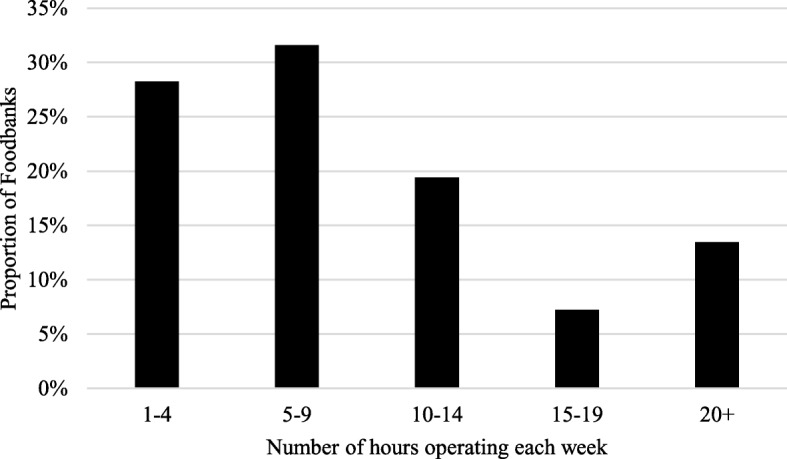
Proportion of Trussell Trust Foodbanks by numbers of hours of operation each week. Note: Information on hours missing for 6 of 392 Foodbanks

Summing distribution centre hours across Foodbanks, the total number of hours that Foodbanks operated across distribution sites was quite low (Fig. 2). Most Foodbanks were open across all their sites for nine or fewer hours each week.

At the local authority level (Additional file 1), on any given weekday, about 30% of local authorities with food banks did not have one open, and among those that did, most only had food banks open for between one to four hours. Only 13.5% of local authorities had food banks that operated in evening hours (Table 1). About 1 in 5 local authorities had a food bank open on Saturdays, but then, only for one to four hours. On Sundays, almost no local authorities had a food bank open (96%).

The average rate of food bank use across all local authorities with Trussell Trust food bank in 2015/16 was 2.37 instances of parcels distributed as a percent of the local area population. Based on the results from our linear regression models, the bivariate relationships between operational characteristics and volume of food bank use at the local authority level are shown in Table 2. For every additional weekday hour that food banks were open in local authorities each week, instances of people receiving food parcels increased by 0.03 (95% CI: 0.019–0.04) per 100 people. On average, instances of people receiving food parcels was 0.54 higher per 100 people in local authorities with food banks open on weekends compared to those with none operating on weekends (95% CI: 0.05–1.03), and for every additional distribution centre operating in local authorities, instances of people receiving food parcels rose by 0.15 (95% CI: 0.10–0.19). There was a non-significant negative relationship between the land area of local authorities and instances of people receiving food parcels (per additional 100 km^2^, food bank use declined by 0.007 per 100 (95% CI: − 0.02 to 0.004), but expressed as a density, instances of people receiving food parcels statistically significantly increased by 0.10 per each additional distribution centre per 100 km^2^ (95% CI: 0.06–0.14). Together, these findings confirm our hypothesis that accessibility of food banks is positively associated with the number of food parcels distributed.

**Table 2:** Bivariate associations of food bank operational characteristics and indicators of need with instances of people receiving food parcels as percent of local area population (2015/16)

	B-coefficient (SE)
*Operational characteristics*	
Per every additional hour food banks open on weekdays	0.030^***^ (0.0054)
Food banks open in evenings	
No	Referent
Yes	0.22 (0.30)
Food banks open on weekends	
No	Referent
Yes	0.54^*^ (0.25)
Per additional distribution site operating	0.15^***^ (0.025)
Per additional 100 KM^2^ of local area size	−0.0072 (0.0056)
Per additional distribution site per 100 KM^2^	0.10^***^ (0.020)
*Indicators of need in population*	
Households receiving Working Tax Credit (% households)	0.081* (0.032)
Disability rate (% working-age adults)	0.096*** (0.022)
Unemployment rate (% working-age adults)	0.161** (0.055)

Notes: Standard errors in parentheses. ^*^ *p* < 0.05, ^**^ *p* < 0.01, ^***^ *p* < 0.001

**Fig. 3:**
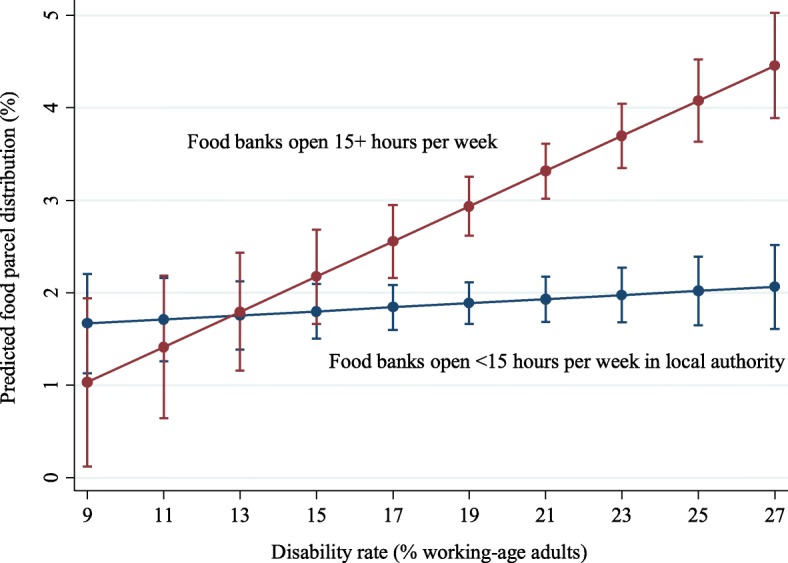
Interaction between disability rate and hours of operation in local authorities on food bank usage

Our linear regression results also show that in-work poverty, disability, and unemployment rates were all associated with a higher number of instances of people receiving food parcels (Table 2). The number of hours that food banks were open during the day and the density of food banks in local authorities both significantly interacted with disability rates. Where disability rates were below 17%, there was no difference in predicted food parcel distribution between local authorities with food banks open fewer than 15 h per week and those with food banks open 15 or more hours (Fig. 3). In areas with higher disability rates, however, predicted food parcel distribution was significantly higher in local authorities with food banks operating at least 15 h per week.

**Fig. 4:**
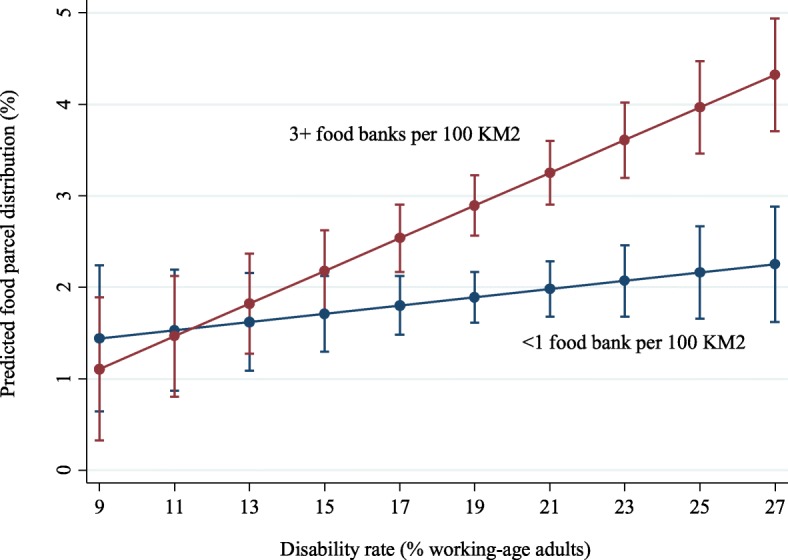
Interaction between disability rate and density of distribution sites in local authorities in relation to food bank usage

A similar pattern was observed for density of food banks (Fig. 4). Instances of people receiving food parcels rose rapidly with increasing disability rates where more than three food banks were operating per km^2^, but the rise was statistically significantly less for areas where there was less than one food bank per km^2^.

The association for rates of disability, then, partly confirms our second hypothesis; however, contrary to our second and third hypotheses, these operational characteristics did not significantly interact with in-work poverty rates or unemployment rates. Specifically, the relationship between food bank usage and in-work poverty was not modified by whether or not food banks were open on evenings or weekends nor by the density of food banks. The relationship between unemployment and food bank usage was also not modified by the density of food banks or number of hours food banks were open (see Additional file 1).

## Discussion

In our examination of one food bank network operating in Britain, which makes up about 60% of all food banks operating, we found food banks are open for only a limited number of hours each week and that there was a relatively low density of food bank distribution sites. These features of access were associated with food bank use, where areas with fewer operating hours and fewer distribution sites per km^2^ served significantly fewer people. Importantly, these characteristics interacted with disability rates, a risk factor for food bank use and food insecurity in Britain [33, 36]. In places with high levels of disability, food bank use was significantly lower where there were fewer food banks and fewer opening hours. A positive relationship between in-work poverty and food bank use was observed but this relationship did not differ by operational characteristics--in particular, whether or not food banks were open on weekends.

These data support earlier research that has raised questions about how effectively food banks reach people at risk of food insecurity, given the absence of accountability of charitable food aid provisioning, barriers to access formed by eligibility thresholds, limitations and referral processes, and logistical issues including distance to food banks and opening times [13]. While patterns of food banks opening suggest they have been more likely to open in places where there have been reductions in social and welfare spending and in areas of high child poverty [17, 27], this study suggests that even if a food bank is present, it does not mean it is accessible, as operating hours may be limited. In-depth research on food aid providers have shown how often the focus is on the practical aspects of providing food to people who *reach* food banks [13, 37], with little time put toward understanding the scale of local need and accessibility of their services.

It was not observed that a lack of food bank hours on weekends diminished the relationship between in-work poverty and food bank usage. This was surprising, as it was one hypothesised reason for why there are few people experiencing in-work poverty among food bank users in the UK [33]. However, research in other countries has suggested that low-income households in work are especially averse to using food charity [38, 39]. People with employment may also be less likely to be in contact with Trussell Trust referral partners. Alternatively, in-work poverty is often characterised by employment in industries with non-standard working hours and part-time working in Britain [40], so it is possible that our observation that most food banks are not open during non-standard working hours is not a barrier to food bank use for the working poor.

### Strengths and limitations

To our knowledge, this is the first quantitative examination of how operational features of food banks correlate with food bank usage. One strength of this study is that it makes use of novel data routinely and consistently collected in The Trussell Trust Foodbank Network. Their harmonisation of data collection across their large network of food banks means that studying patterns of usage by operational characteristics is possible.

This analysis is limited, however, in that only measures of operation in three dimensions were captured: when food banks were open, how long they were open for, and the density of distribution sites. There are other features of how food banks operate that could restrict access, including the number of referring agencies, how strict referring agencies are in making referrals, how accessible referring agencies are, and the nature of referring agencies themselves. For example, referring agencies include Jobcentre Plus offices, which may be more likely to see people who are unemployed or receiving disability benefits than people who are in work. Importantly, referral agencies may have their own eligibility criteria for providing food bank vouchers, but to our knowledge, these have not been explored. Another critical aspect is how often people can receive referrals to food banks. The Trussell Trust provides guidance to their member food banks that they should enquire if a referring agent provides more than three referrals in a six-month period. Future research is needed to explore these many dimensions of food bank access. However, studies have also shown that other factors, such as stigma and not wanting to receive help from a charity, also influence who, among people experiencing food insecurity, use food banks [38]. These are also critical factors to explore with regard to how adequately food banks are able to meet the needs of people experiencing food insecurity.

This study is also limited because it relies solely on data from The Trussell Trust. While Trussell Trust food bank distribution centres make up about 60% of food banks in the UK, over 800 independent food banks operate weekly that are not members of The Trussell Trust [31]. Operational data may not be generalizable to food banks outside of The Trussell Trust. However, these findings are consistent with those that have characterised food bank operations in Canada, which found that regardless of whether or not food banks were part of a national network, they shared similar operational limitations [25]. Future research should examine the intersection of Trussell Trust and other food banks and their operations to understand the full scale, and potential limitations, of food banks operating in the UK. Another limitation of the data is that it is cross-sectional and observational data; therefore, though our data show an association between operational characteristics and food bank usage, we cannot conclude that this relationship is causal.

### Implications

These findings raise questions about the ability of food banks to address food insecurity. We found operational characteristics are associated with food bank usage, suggesting that the ability of people who are food insecure to receive help from food banks is contingent upon how accessible this help is. Of particular concern is that operational characteristics appear to alter the link between need and food bank usage. While efforts could be made to expand the numbers of hours that food banks operate, when they operate, or their availability, because volunteer labour is so intrinsic to food bank models in the UK [30], the ability to implement expanded operations is questionable. Even in Canada, where food banks have been operating for over 30 years, a recent analysis of operations highlighted that most had limited operating hours and capacity [25]. As has been explored in the United States [22] and Canada [25, 41], inherent features of charitable responses to hunger often restrict their effectiveness. Public policy interventions are needed to address hunger [42].

Though from a practice perspective, these data suggest that food banks should coordinate hours across local catchment areas and that local needs assessments should be made to understand their reach in their communities, even if these efforts were made to improve access, other studies have shown that the limited quality and quantity of food available from food banks is unlikely to address the food insecurity and nutritional needs of the populations that access them [20, 23, 24, 42].

This study also shows that food bank operational characteristics are associated with how many people use food banks. This is problematic in the UK because media and policymakers often rely on Trussell Trust food bank usage as an indicator of whether hunger is increasing. This analysis shows need can vary across the country but might not be reflected in demand for food banks where food banks are less accessible. Regular monitoring of household food insecurity in the UK is needed to understand this critical public health issue.

## Conclusions

As more frontline professionals are given information about food banks and encouraged to provide referrals in the UK, a key question is whether these professionals are referring people in need to services that are accessible and responsive to their need. This study suggests that food banks may not be available and accessible, and these features may affect how closely need is linked to usage. In light of these findings and other studies highlighting the limitations of food banks across other dimensions, including limited food quantity and quality [20, 24] and the socially inappropriate nature of receiving food charity [38], there is an urgent need for better responses to food insecurity in the UK. Evidence suggests public policy responses to address household food insecurity will likely be most effective [42].

## Additional file

Additional file 1: Additional Tables and Figures. (DOCX 101 kb)

## Abbreviations

GP: General practitioner

## Footnotes

1 We report these data as a proportion of the population to adjust numbers for population size.
