# Supplementary material for: Food bank operational characteristics and rates of food bank use across Britain
Source: BMC Public Health. 2019 May 14;19:561. doi: 10.1186/s12889-019-6951-6 (PMC6518699; doi:10.1186/s12889-019-6951-6)
Supplement: Supplementary file 1 — Additional Tables and Figures. (DOCX 101 kb) [file 12889_2019_6951_MOESM1_ESM.docx]

**Web Appendix *to*** Food bank operational characteristics and rates of food bank use across Britain.

**Figure A1** Analytic sample Trussell Trust Foodbanks and local authorities.


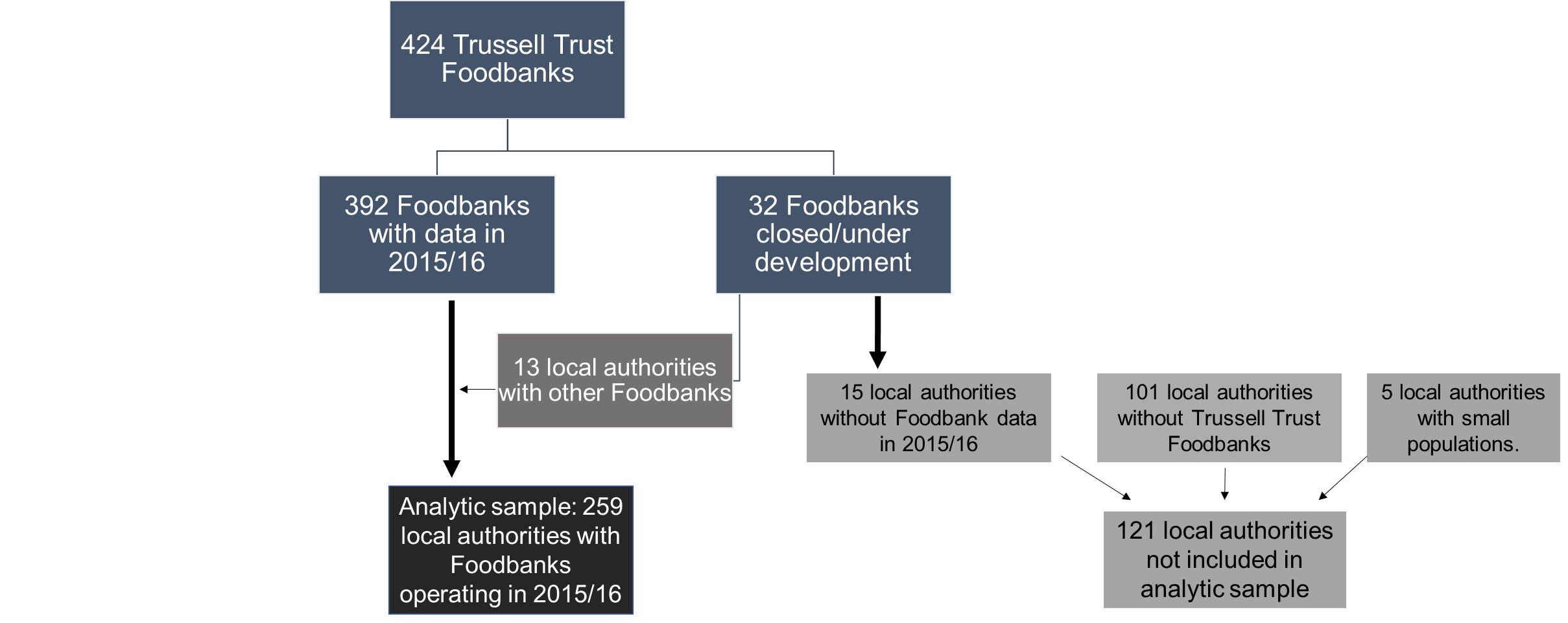


**Figure A2** Days of the week that food banks operate and number of hours across local authorities with food banks (n=259).

**Table A1** Association between instances of people receiving food parcels as percent of local area population (2015/16) and interactions of food bank operations with working-tax credits.

|  |  | B-coefficient (SE) | | | |
| --- | --- | --- | --- | --- | --- |
|  |  | (1) | (2) | (3) |  |
| Households receiving Working Tax Credit (% households) | | 0.089^*^ (0.04) | 0.063 (0.03) | 0.15^**^ (0.06) |  |
| Food banks open on weekends | |  |  |  |  |
|  | No | Referent |  |  |  |
|  | Yes | 1.38 (0.94) |  |  |  |
| Interaction term with Working Tax Credits | | -0.080 (0.08) |  |  |  |
| Food bank daytime opening hours | |  |  |  |  |
|  | Less than 15 hours per week |  | Referent |  |  |
|  | 15 hours + per week |  | 0.72 (0.77) |  |  |
| Interaction term with Working Tax Credits | |  | 0.056 (0.07) |  |  |
| Food bank density | |  |  |  |  |
|  | Less than 1 per 100 KM^2^ |  |  | Referent |  |
|  | 1-2 per 100 KM^2^ |  |  | 2.45^*^ (0.95) |  |
|  | 3+ per 100 KM^2^ |  |  | 2.11^*^ (0.86) |  |
| Interaction terms with Working Tax Credits | |  |  |  |  |
|  | 1-2 per 100 KM^2^ |  |  | -0.18^*^ (0.08) |  |
|  | 3+ per 100 KM^2^ |  |  | -0.11 (0.08) |  |
| Number of local authorities | | 257 | 254 | 257 |  |

Standard errors in parentheses. ^*^ *p* < 0.05, ^**^ *p* < 0.01, ^***^ *p* < 0.001. Constant not shown.

**Table A2** Association between instances of people receiving food parcels as percent of local area population (2015/16) and interactions of food bank operations with disability rate.

|  |  | B-coefficient (SE) | | |
| --- | --- | --- | --- | --- |
|  |  | (1) | | (2) |
| Disability rate (% working-age adults) | | 0.022 (0.02) | 0.045 (0.04) | |
| Food bank daytime opening hours | |  |  | |
|  | Less than 15 hours per week | Referent |  | |
|  | 15 hours + per week | -2.15^*^ (0.93) |  | |
| Interaction term with disability rate | | 0.17^***^ (0.05) |  | |
| Food bank density | |  |  | |
|  | Less than 1 per 100 KM^2^ |  | Referent | |
|  | 1-2 per 100 KM^2^ |  | 0.55 (1.10) | |
|  | 3+ per 100 KM^2^ |  | -1.54 (1.01) | |
| Interaction terms with disability rate | |  |  | |
|  | 1-2 per 100 KM^2^ |  | -0.0031 (0.05) | |
|  | 3+ per 100 KM^2^ |  | 0.13^**^ (0.05) | |
| Number of local authorities | | 254 | 257 | |

Standard errors in parentheses. ^*^ *p* < 0.05, ^**^ *p* < 0.01, ^***^ *p* < 0.001. Constant not shown.

**Table A3** Association between instances of people receiving food parcels as percent of local area population (2015/16) and interactions of food bank operations with unemployment rate.

|  |  | B-coefficient (SE) | | |
| --- | --- | --- | --- | --- |
|  |  | (1) | (2) |  |
| Unemployment rate (% working-age adults) | | 0.075 (0.07) | 0.24^*^ (0.10) |  |
| Food bank daytime opening hours | |  |  |  |
|  | Less than 15 hours per week | Referent |  |  |
|  | 15 hours + per week | 0.88 (0.61) |  |  |
| Interaction term with unemployment rate | | 0.079 (0.11) |  |  |
| Food bank density | |  |  |  |
|  | Less than 1 per 100 KM^2^ |  | Referent |  |
|  | 1-2 per 100 KM^2^ |  | 0.96 (0.77) |  |
|  | 3+ per 100 KM^2^ |  | 2.26^**^ (0.75) |  |
| Interaction terms with working tax credits | |  |  |  |
|  | 1-2 per 100 KM^2^ |  | -0.12 (0.15) |  |
|  | 3+ per 100 KM^2^ |  | -0.27 (0.14) |  |
| Number of local authorities | | 230 | 233 |  |

Standard errors in parentheses. ^*^ *p* < 0.05, ^**^ *p* < 0.01, ^***^ *p* < 0.001. Constant not shown.
